# Hard and soft tissue evaluation of alveolar ridge preservation compared to spontaneous healing: a retrospective clinical and volumetric analysis

**DOI:** 10.1186/s40729-022-00456-w

**Published:** 2022-12-08

**Authors:** Paolo De Angelis, Giuseppe De Rosa, Paolo Francesco Manicone, Alessandro De Giorgi, Camilla Cavalcanti, Alessandro Speranza, Roberta Grassi, Antonio D’Addona

**Affiliations:** 1 Division of Oral Surgery and Implantology, Department of Head and Neck, Institute of Clinical Dentistry, Oral Surgery and Implantology Unit, Fondazione Policlinico Universitario A. Gemelli IRCCS-Università Cattolica del Sacro Cuore, 00168 Rome, Italy; 2 Polytechnic University of Turin, Turin, Italy; 3 Private Practice, Rome, Italy; 4 Department of Oral Surgery, Tor Vergata University, 00133 Rome, Italy

**Keywords:** Alveolar ridge preservation, Bone graft, Tooth extraction

## Abstract

**Purpose:**

The remodeling process following tooth extraction can be observed as horizontal and vertical bone reduction of the alveolar ridge. Preservation procedures such as alveolar ridge preservation (ARP) aim to maintain the 3D volume of the extraction site. This retrospective study analyzed differences in the hard and soft tissue changes in patients treated with either spontaneous healing or ARP.

**Methods:**

After tooth extraction, the patients were treated either by spontaneous socket healing (SH group) or with ARP using a xenograft and a resorbable membrane (ARP group). One week before and 6 months after extraction, the patients underwent cone beam computed tomography. A volumetric analysis was performed by superimposing the digital models of the two time points. Intraoral radiography was performed after implant placement, upon prosthesis delivery, and at 1-year post-treatment. An esthetic assessment was conducted using the Pink Esthetic Score (PES). The patients’ overall satisfaction with the implant restoration was investigated at 12 months.

**Results:**

Intragroup comparisons revealed significant differences between baseline and the 6-month follow-up in both groups at the measured locations (1 mm, 3 mm, and 5 mm below the most coronal aspect of the alveolar ridge) showing a reduction of the horizontal width (*P* < 0.05). Additionally, after treatment, the horizontal width at 1 mm was significantly different in the SH and ARP groups (*P* < 0.001), with mean changes of 2.03 ± 0.54 mm and 0.86 ± 0.49 mm, respectively. ARP was associated with an increased PES (11.6 ± 2.2) and a reduction in patients requiring additional grafting procedures in subsequent treatment phases (9% vs 26%; *P* = 0.11).

**Conclusions:**

In both groups, significant horizontal and vertical bone loss was observed after the extraction. ARP can reduce linear and volumetric shrinkage of the alveolar ridge, leading to improved outcomes. It can also simplify implant restoration.

**Graphical Abstract:**

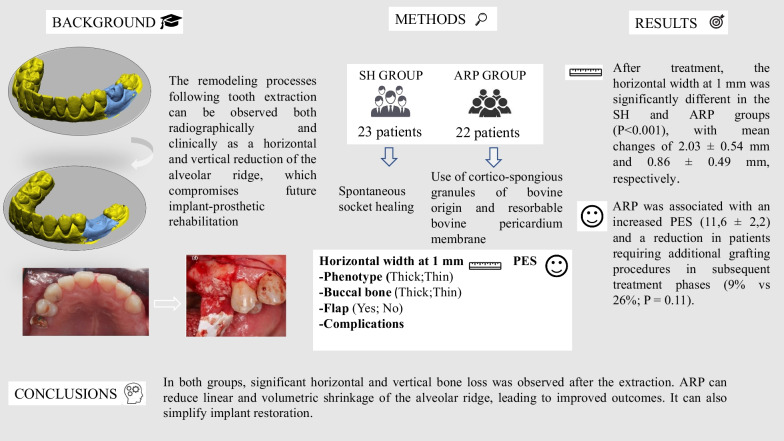

## Background

Extraction of a compromised tooth is indicated in cases where restoration cannot be guaranteed by conservative approaches [1].

The alveolar process is a tooth-dependent tissue. Its shape and volume are determined by several factors such as tooth anatomy and the axis of eruption and inclination. The loss of a tooth results in bone resorption of the alveolar process and the bundle bone, which is the portion of the alveolar process that surrounds the teeth and into which the collagen fibers of the periodontal ligament are embedded [2, 3].

The remodeling processes following tooth extraction can be observed both radiographically and clinically as a horizontal and vertical reduction of the alveolar ridge, which compromises future implant-prosthetic rehabilitation [4, 5].

Alveolar ridge preservation (ARP) strategies aim to minimize the loss of ridge volume that occurs during bone remodeling. ARP was introduced in the 1980s and, due to the predictability of the technique, has gained popularity over the years [6].

The potential advantages of ARP include the maintenance of soft and hard tissue volume, which optimizes functional and esthetic results and creates favorable conditions for subsequent treatment procedures [7]. There are various clinical protocols for ARP. Bone grafts (autografts, allografts, xenografts, and alloplastic materials) can be used with resorbable or non-resorbable barriers, soft tissue grafts, or biologically active materials (growth factors) [8] to reduce the loss of alveolar bone height and width.

The use of ARP techniques delays implant placement by at least 4 to 6 months, depending on the approach and biomaterials used [9, 10], with longer treatment times than immediate and early implant placement.

Tonetti et al. compared spontaneous healing of the post-extraction alveolus with ARP and found that 1.5–2.4 mm of horizontal alveolar ridge resorption, 1–2.5 mm of vertical buccal resorption, and 0.8–1.5 mm of vertical lingual resorption can be prevented by using ARP [11]. More bone resorption occurs on the buccal side of the alveolar ridge than on the lingual/palatal side, resulting in a dimensional shift of the ridge to a more lingual/palatal position [12].

The volume reduction after spontaneous healing of the alveolar ridge may lead to the patient requiring bone regeneration procedures at the same time or prior to implant placement. However, extraction sites treated with alveolar ridge preservation techniques seem to reduce the need for additional bone grafting in the subsequent clinical phases and can also simplify the prosthetic rehabilitation, allowing prosthetically guided implant positioning [13].

In recent years, several studies have evaluated which type of graft is most suitable among autologous bone, allografts, xenografts, and alloplastic materials [9]. These materials have shown similar results when used in ARP. In particular, the use of a xenograft or allograft is associated with better results in terms of alveolar ridge preservation [14].

The purpose of this study is to test the null hypothesis of no difference in the alveolar ridge and soft tissue changes between spontaneous healing and ARP by performing linear and volumetric measurements and investigating the factors affecting the outcomes. The primary outcomes of the study are the vertical and horizontal changes of the alveolar ridge measured at the 6-month follow-up. The secondary outcomes are the volumetric analysis of the soft tissue, the pain perceived by the patients and the PES.

## Methods

This was a retrospective comparative study to evaluate the alveolar ridge changes after a single dental extraction and the outcomes of the prosthetic rehabilitation by comparing spontaneous healing with ARP performed prior to implant placement.

Participants in this study were recruited from the Division of Oral Surgery and Implantology of the Catholic University of the Sacred Heart of Rome between January 1 and June 31, 2020. The study was conducted in full compliance with the ethical principles expressed in the Declaration of Helsinki (2008).

The treatment was made after a preoperative clinical and radiographical evaluation and a discussion with the patient. All patients were informed about the benefits and risks of undergoing ARP based on the current scientific evidence and the individual treatment plan. Patients who refused ARP were treated in accordance with the spontaneous healing protocol.

The inclusion criteria for the study were:

- Age > 18 years;
- Ability to sign an informed consent form;
- Need for a single-tooth extraction;
- Need for implant-prosthetic rehabilitation;
- Sufficient vertical and horizontal bone dimensions to receive an implant-supported restoration;
- Presence of an intact post-extractive alveolus

The exclusion criteria for the study were:

- General contraindications to surgery;
- Assumption of long-term nonsteroidal anti-inflammatory therapy;
- Intake of bisphosphonate medications;
- History of radiation therapy of the head and neck area within the past 5 years;
- Uncontrolled metabolic disorders;
- Uncontrolled periodontal disease;
- Pregnancy and lactation;
- Presence of sites with acute infection that could not be resolved at the time of extraction (e.g., abscess, phlegmon);
- Absence of dental occlusion in the arch opposite the area of the extraction site;
- Cigarette consumption > 10 per day;
- Reluctance to undergo follow-up visits during the follow-up period.

After tooth extraction, the alveolus was carefully cleaned and inspected using a periodontal probe and treated using one of the following clinical protocols:

- Spontaneous healing (SH) group: spontaneous socket healing
- Alveolar ridge preservation (ARP) group: use of cortico-spongious granules of bovine origin (Re-Bone, UBGEN, Padova, Italy) and resorbable bovine pericardium membrane (Shelter Fast, UBGEN).

Patient baseline factors were assessed before the tooth extraction (T0) and were retrieved from the patients’ archives and clinical charts. The factors considered were the facial soft tissue thickness (FSTT) and the preoperative buccal bone thickness. The FSTT evaluation, recorded in the clinical chart, was performed preoperatively 1 mm apical from the mid-buccal point located on the mucosal margin by probing with an endodontic file before the extraction and after the anesthesia. FSTT was classified as thick or thin: tissue thickness greater than 1 mm was categorized as thick; tissue thickness less than 1 mm was categorized as thin. The preoperative buccal bone thickness was assessed using cone beam computed tomography (CBCT) and categorized as thick or thin.

The patients underwent CBCT (Pax-i3D Smart, Vatech, Hwaseong, Korea) before surgery and 6 months after extraction. The data were retrieved and converted to DICOM (Digital Imaging and Communications in Medicine) format and imported into an open-source software for 3D image processing. The DICOM files of each patient at baseline and at 6 months post-extraction were imported into the software and overlaid using anatomical landmarks where no changes occurred during the follow-up period—for example, the palatine vault, the anterior or posterior nasal spine, or the lower angle border of the mandible.

Linear measurements on the CBCT images were performed as previously described by Jung et al. (2013) [15] at baseline (T0) and at 6 months (T1) using the same reference points and lines. A vertical reference line was drawn at the center of the socket, crossing the apical landmark, which is the most apical point of the extractive socket defined on preoperative CBCT images. Two horizontal reference lines were drawn perpendicular to the vertical line, traversing the most coronal portion of the palatal/lingual bone crest and the most apical point of the extractive socket. The following parameters were reported (Fig. 2):

- Buccal (BH) and palatal (PH) bone ridge height at baseline and at the 6-month follow-up;
- The horizontal width of the alveolar ridge measured at 1 mm, 3 mm, and 5 mm below the most coronal aspect of the alveolar ridge at baseline and at 6 months (HW-1, HW-3, HW-5);
- Preoperative buccal bone thickness at 1 mm (BBT-1) (at baseline only).

### Volumetric analysis of the soft tissue

To analyze changes in the soft tissue volume in the ARP and SH groups, a preoperative digital model of the dental arches, used preoperatively for planning the rehabilitation treatment, was compared with the 6-month follow-up for each patient. The data obtained were exported as STL files and imported into a software for processing.

For each patient, the digital model obtained at baseline was superimposed with the one obtained at the follow-up to examine volumetric changes. The models were superimposed with fiducial registration. The preoperative (T0) model was used as the reference, and that obtained at follow-up (T1) was used as the moving model. Before registration, the same landmarks were marked on the dental elements of both models to provide registration input to the program. Once the models were superimposed, a manual check was performed to verify the match and correct any discrepancies.

To perform the volumetric measurement, a region of interest was identified on the preoperative model, extending in the mesio-distal direction from the mesial and distal interdental papilla of the tooth that had to be extracted and in the apico-coronal direction from the most apical point (10 mm from the gingival margin) to the gingival margin. The overlapping models were segmented accordingly.

Once isolation of the region of interest was obtained. The follow-up image was subtracted from the preoperative image to obtain an image showing the net change in ridge volume excluding the volume of the crown.

Finally, the obtained segment was analyzed through Geomagic software to calculate its volume, thus obtaining a value that represents the difference in soft tissue volume in the region of interest between the preoperative and postoperative models.

During each step of the process, the segments obtained were carefully compared with the original models for case verification. In the final step, they were superimposed onto the phantom of the preoperative model for visual analysis of the soft tissue change.

After implant placement, intraoral radiography using the long cone parallel technique was performed at prosthesis delivery and 1 year post treatment, as required by the department’s protocol. A bite made of silicone (3 M™ Express, 3 M ESPE Dental Products, St. Paul, USA) was placed on the holding system, allowing the clinician to reposition it precisely during each follow-up visit. Linear measurements (mm) on the digital images were obtained to record the distance of the most coronal point from the implant shoulder.

The pain perceived by the patients was recorded in the clinical chart and evaluated with a self-developed visual analog scale (VAS) questionnaire filled out by the patients after 7 days from the surgery.

An esthetic assessment was performed using the Pink Esthetic Score (PES) 1 year post treatment. The PES was retrieved from the chart as well as the patient-reported outcomes, which were evaluated with a self-developed visual analog scale (VAS) questionnaire filled out by the patients. The VAS was used at 12 months to assess the patients’ overall satisfaction with the implant restoration.

### Surgical procedure

Before initiating the treatment plan, the patients underwent periodontal procedures to establish an adequate oral hygiene condition. All patients rinsed with a 0.2% chlorhexidine mouthwash (Curasept ADS, Curasept S.p.A., Saronno, Italy) for 1 min, and the surgical area was anesthetized using an infiltrative technique (4% articaine, 1: 100,000 with adrenaline, Pierrel S.p.A., Capua, Italy).

A flapless approach, performed as atraumatically as possible using periotomes, was used when indicated; however, a full-thickness muco-periosteal flap was elevated to carry out complex extractions.

A periotome was used around the circumference of the tooth to sever the attachment of the coronal periodontal fibers. The tooth was extracted with the use of fine levers and pliers, paying particular attention to avoid bucco-lingual movements to prevent damage or fracture of the buccal cortical. If necessary, the tooth was sectioned with a diamond bur to facilitate extraction. Subsequently, the alveolus was gently curetted to remove all of the granulomatous tissue and irrigated with sterile saline. The surgeon also debrided the adjacent teeth. A resorbable suture (4-0 PGA, Perma Sharp Sutures, Hu-Friedy, Chicago, USA) was used to stabilize the wound margins, which were left to heal by secondary intention.

**Fig. 1:**
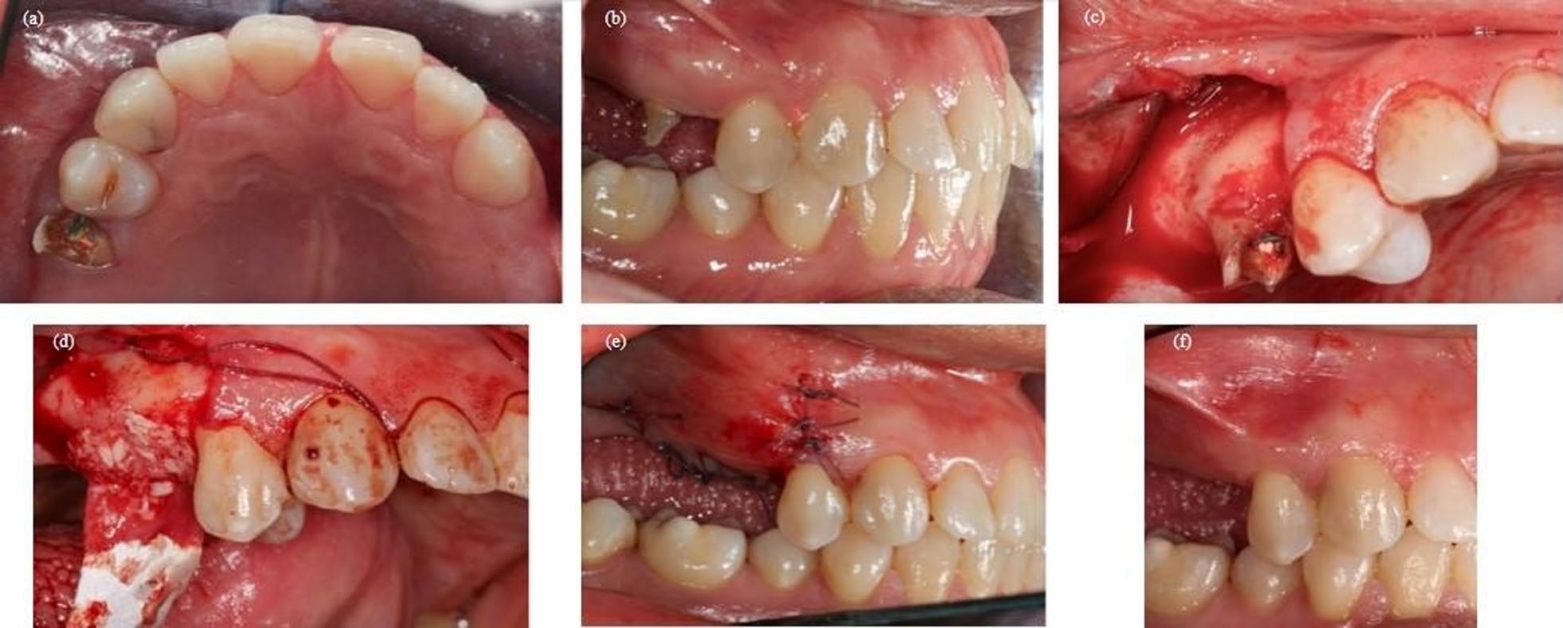
Clinical case treated following the alveolar ridge preservation protocol. **a** Occlusal view of the fractured tooth. **b** Buccal view of the fractured tooth. **c** Mucoperiosteal flap elevation. **d** Post-extractive alveolus filled with the bone xenograft and covered with the resorbable membrane. **e** Suture of the surgical site. **f** Post-operative healing of the treated area

**Fig. 2:**
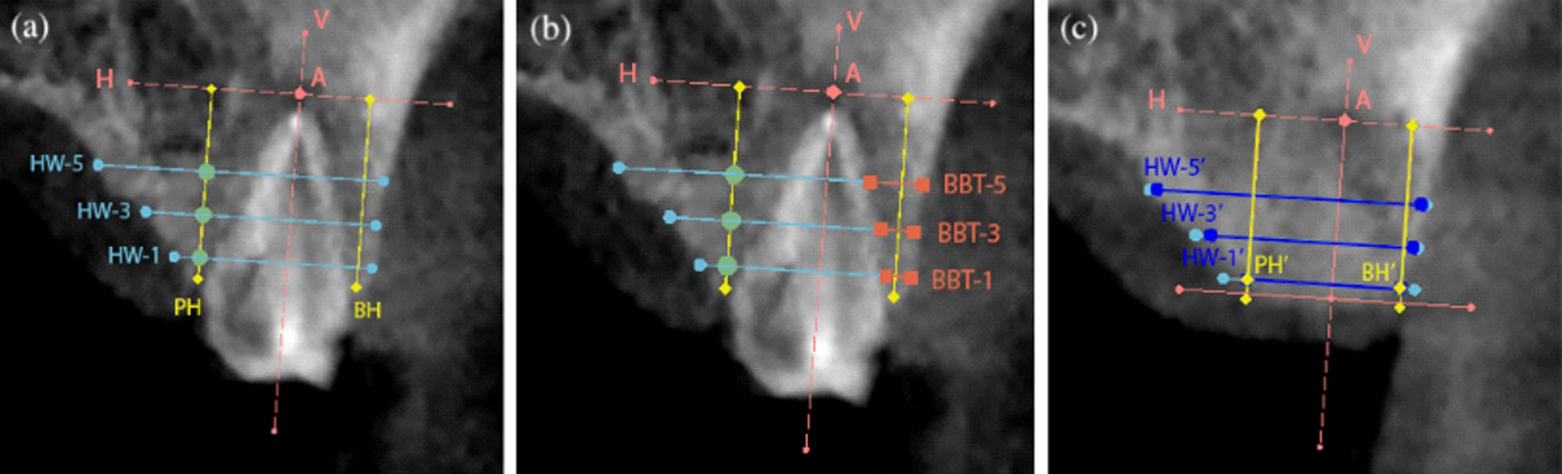
Linear measurements on CBCT images of the case treated following the alveolar ridge preservation protocol. **a**) Preoperatively the horizontal analysis was performed at 1, 3 and 5 mm (HW-1, HW-3, HW-5) and the vertical analysis was performed on the buccal and palatal side (BH, PH); **b**) preoperatively the buccal bone thickness was measured at 1, 3 and 5 mm (BBT-1, BBT-3, BBT-5); **c**) postoperatively the horizontal analysis was performed at 1, 3 and 5 mm (HW-1, HW-3, HW-5) and the vertical analysis was performed on the buccal and palatal side (BH, PH)

**Fig. 3:**
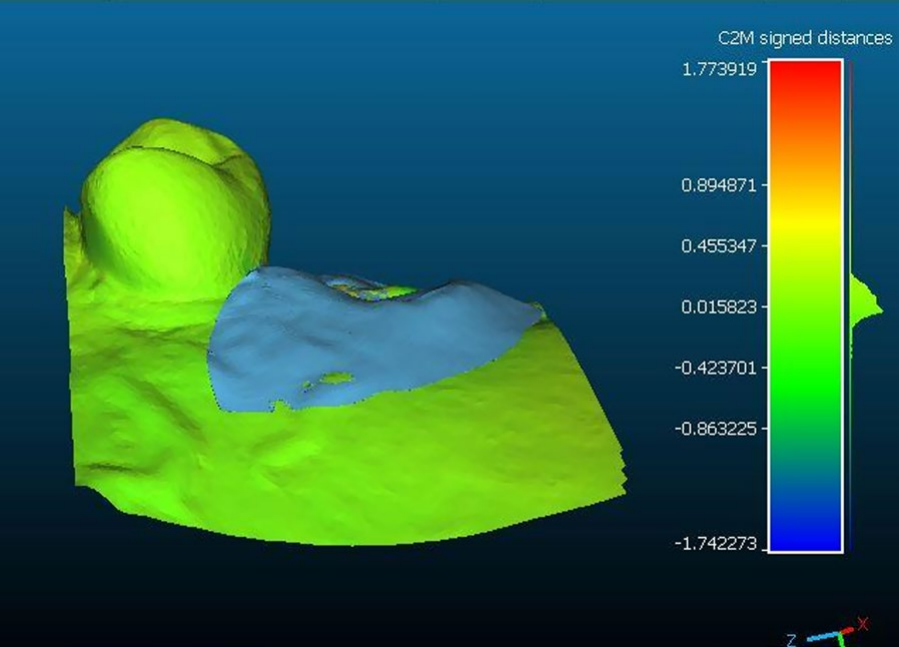
Buccal view showing the change of the soft tissue volume in a case treated following the alveolar ridge preservation protocol

**Fig. 4:**
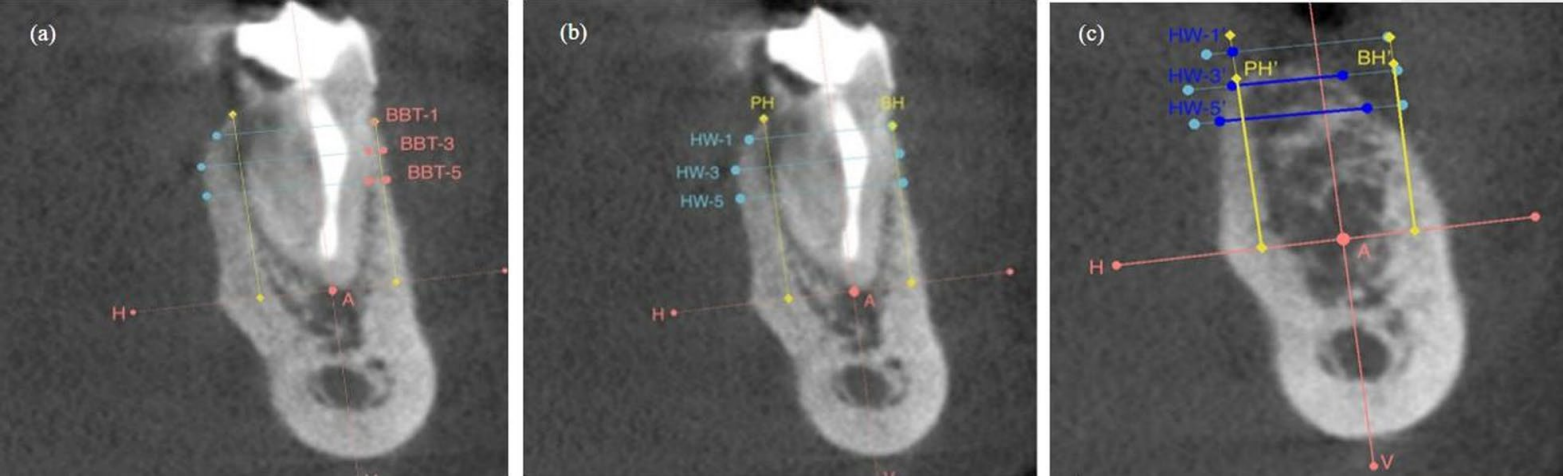
Linear measurements on CBCT images of the case treated following the spontaneous healing protocol. **a**) Preoperatively the horizontal analysis was performed at 1, 3 and 5 mm (HW-1, HW-3, HW-5) and the vertical analysis was performed on the buccal and palatal side (BH, PH); **b**) preoperatively the buccal bone thickness was measured at 1, 3 and 5 mm (BBT-1, BBT-3, BBT-5); **c**) postoperatively the horizontal analysis was performed at 1, 3 and 5 mm (HW-1, HW-3, HW-5) and the vertical analysis was performed on the buccal and palatal side (BH, PH)

**Fig. 5:**
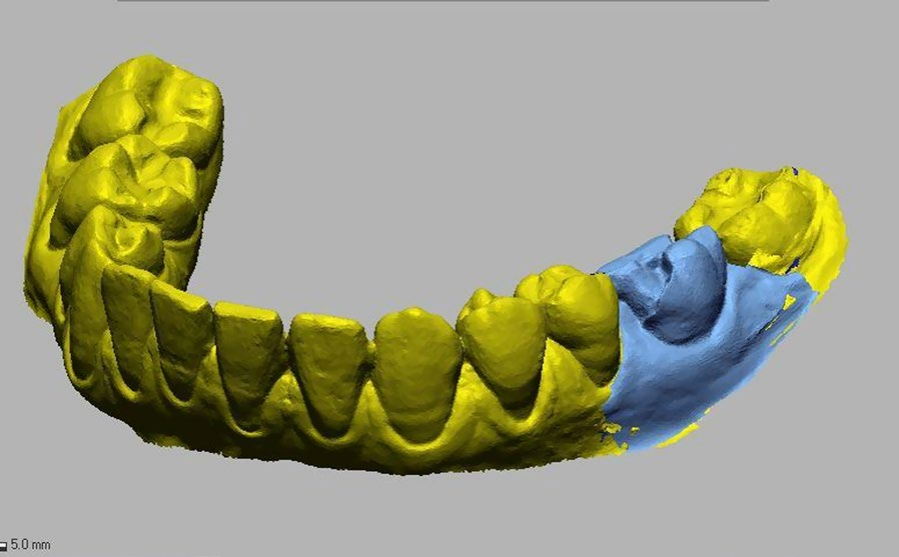
Buccal view showing the change of the soft tissue volume in a case treated following the spontaneous healing protocol

**Fig. 6:**
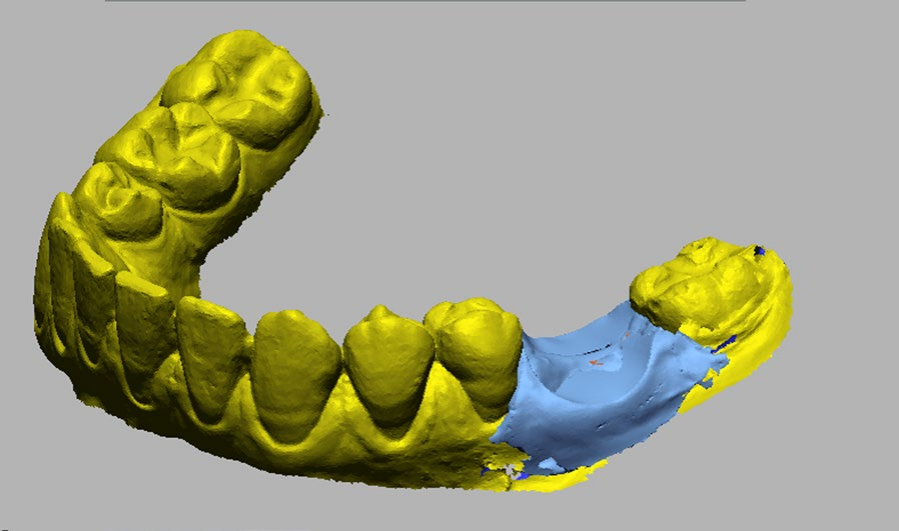
Buccal view showing the change of the soft tissue volume after tooth segmentation

In the ARP group, the alveolus was packed with cortico-spongious granules of bovine origin that were processed at low temperatures to preserve bone porosity and avoid ceramization. The granules were slightly condensed until filling the entire alveolus and reaching the coronal margin of the alveolar bone. The particles were 0.25 to 1 mm in size. The graft was covered with a resorbable bovine pericardium membrane placed under the buccal and lingual/palatal flap. Compressive sutures with a resorbable suture (4–0 PGA, Perma Sharp Sutures, Hu-Friedy) were then used to stabilize the graft (Figs. 1, 2, 3, 4, 5, 6).

All patients were given verbal and written postoperative instructions and instructed to rinse their mouth with 0.12% chlorhexidine mouthwash twice per day for 14 days. An antibiotic (amoxicillin, 1 g every 12 h for 6 days) and an anti-inflammatory drug (Ketoprofen 50 mg at 12 h intervals for 3 to 4 days) were prescribed to all participants. The patients were re-examined after 7 days for a follow-up visit and again 14 days after the extraction for suture removal. The patients were followed up again at 6 weeks and 6 months after extraction. The implant placement was carried out with a second surgery performed after the 6-month follow-up visit. After prosthesis delivery, all patients were engaged in a maintenance program.

### Statistical analysis

Qualitative variables were described as absolute and relative frequencies, while quantitative variables were summarized as mean and standard deviation.

Independent samples *t*-tests were conducted for each variable to assess the intragroup differences from baseline and the differences in the reported outcomes between the SH and ARP groups.

All analyses were performed with STATA software, and a *P*-value of < 0.05 was set as the threshold for statistical significance.

## Results

The study sample consisted of 45 patients (21 women and 24 men; mean age: 50.7 years; SD: 12.61 years). Twenty-three patients (mean age: 49.96 ± 11.32 years; 10 females and 13 males) were included in the SH group, and 22 patients (mean age: 51.05 ± 12.61 years; 11 females and 11 males) were included in the ARP group.

All participants underwent a single dental extraction between January 1, 2019 and January 1, 2020. Reasons for extraction were crown/root fractures (*n* = 16; 36%), trauma (*n* = 3; 7%), destructive carious lesions (*n* = 24; 53%), or external root resorption (*n* = 2; 4%).

From an initial sample of 58 eligible patients, 3 patients moved out of the city, and 10 patients were not available at the follow-up examinations.

All of the surgeries were successfully carried out, and no intraoperative complications were recorded. After the surgery, two patients (9%) in the ARP group showed delayed wound healing but no signs of acute infection and did not require adjunctive therapy.

A thick FSTT (> 1 mm) was observed in 10 patients (22%), while a thin FSTT (≤ 1 mm) was identified in 35 patients (78%) during the preoperative examination. No significant difference in mean FSTT was found preoperatively between the two groups (*P* = 0.91). No significant difference in mean preoperative buccal bone thickness was found between the two groups (*P* = 0.87). In the SH group a thick preoperative buccal bone thickness was observed in 14 patients while a thin one in 9 patients. In the ARP group a thick preoperative buccal bone thickness was recorded in 9 patients while a thin one in 13 patients. In the SH group, 8 patients (35%) underwent extraction in anterior sites and 15 in posterior sites (65%); in the ARP group, 6 patients (27%) underwent extraction in anterior sites and 16 (73%) in posterior sites.

In both groups, the horizontal width of the alveolar ridge decreased from baseline at all of the measured points; the mean values are reported in Table 1. Intragroup comparisons with paired t-tests found significant differences between T0 and T1 in both groups at all of the measured points (HW-1, HW-3, HW-5) showing a reduction of the horizontal width.

**Table 1:** Horizontal width change 6 months after extraction

	Horizontal width at 1 mm (HW-1)	Horizontal width at 3 mm (HW-3)	Horizontal width at 5 mm (HW-5)
SH Group	2*.*03 ± 0*.*54 mm	1*.*35 ± 0*.*50 mm	0*.*94 ± 0*.*46 mm
ARP Group	0*.*86 ± 0*.*49 mm	0*.*55 ± 0*.*42 mm	0*.*46 ± 0*.*37 mm

**Fig. 7:**
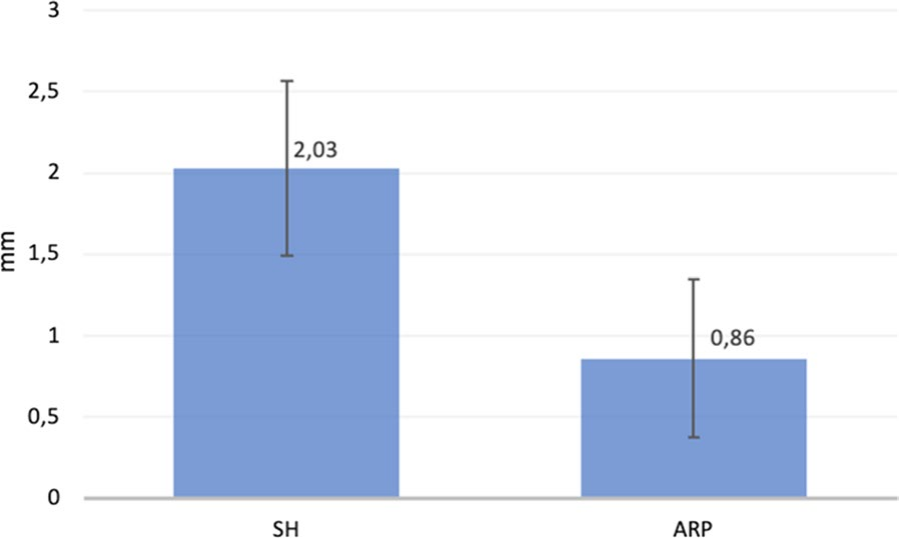
Mean comparison of horizontal bone change at Horizontal Width 1 (HW-1) from baseline

**Fig. 8:**
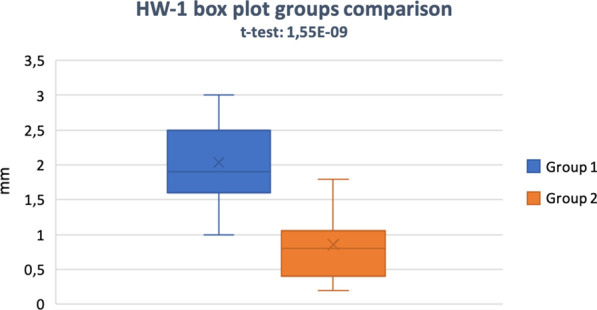
Box plot comparison at Horizontal Width 1 (HW-1)

After treatment, the HW-1 values were significantly different in the SH and the ARP groups, with mean changes of 2.03 ± 0.54 mm and 0.86 ± 0.49 mm, respectively (*P* < 0.001) (Figs. 7, 8).

Significant differences between the two groups were also observed for HW-3 and HW-5 (*P* < 0.001).

Compared with baseline, the volume of the region of interest was significantly reduced postoperatively in both groups (*P* < 0.001). The volumetric analysis revealed that the postoperative volume was significantly different in the SH and ARP groups, with a mean change of 106.41 ± 24.32mm^3^ and 62.66 ± 17.50 mm^3^, respectively (*P* = 0.004).

The *t* test for independent samples results are reported in Tables 2 and 3. In both groups, the preoperative FSTT and the preoperative buccal bone thickness were associated with a significantly lower reduction of HW-1.

**Table 2:** Summary of the variables considered for the t test for independent samples in SH group

Horizontal width at 1 mm SH Group	p-value
*Phenotype*	0.0003
Thick	
Thin	
*Buccal bone*	0.0000346
Thick	
Thin	
*Flap*	0.00004
Yes	
No	
*Complications*	0.24

**Table 3:** Summary of the variables considered for t test for independent samples in ARP group

Horizontal width at 1 mm ARP Group	p-value
*Phenotype*	0.0066
Thick	
Thin	
*Buccal bone*	0.03
Thick	
Thin	
*Flap*	0.22
Yes	
No	
*Complications*	0.0000023

In SH Group the flapless technique was associated with a significantly lower reduction of HW-1 (*P* < 0.001) while in ARP group the occurrence of complications affected negatively the results (*P* < 0.001).

In the SH group, the flap elevation was associated with a larger reduction of HW-1.

**Fig. 9:**
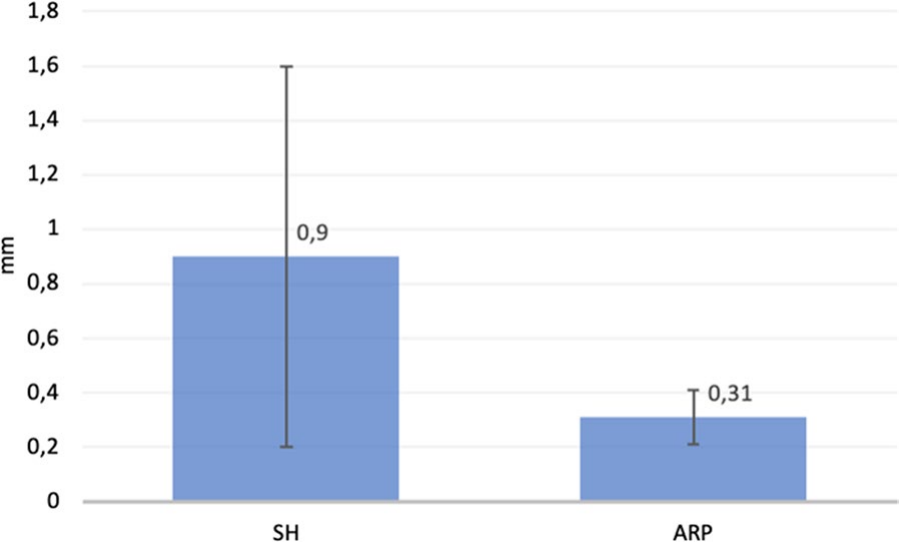
Mean comparison of vertical buccal bone plate change from baseline

In the SH group, the vertical dimensions of the buccal and palatal bone plate decreased from baseline with mean changes of 0.9 ± 0.7 and 0.75 ± 0.04, respectively. In the ARP group, the buccal and palatal vertical changes were 0.31 ± 0.1 and 0.25 ± 0.03, respectively (Fig. 9).

The intragroup and the intergroup comparisons (paired *t*-tests) revealed significant differences between T0 and T1 (*P* < 0.05). There was no significant difference in pain after the procedures (*P* = 0.89). There was a significant difference in PES 1 year after prosthesis delivery (*P* = 0.007); however, patient satisfaction was similar in the two groups (*P* = 0.79).

In the SH group, the mean for PES and patient satisfaction were, respectively, 9.9 ± 1.8 and 8.1 ± 1.1. In the ARP group, the mean for PES and patient satisfaction were, respectively, 11.6 ± 2.2 and 8 ± 1.2.

Additional grafting procedures prior to or during the implant placement were required by two patients (9%) in the ARP group (transcrestal sinus lift) and by 6 patients (26%) in the SH group (*P* = 0.11).

There were no instances of peri-implantitis with pathologic and progressive measurements of the peri-implant marginal bone loss in either group.

All participants received their planned rehabilitation treatment, with a 1-year implant survival rate of 100%.

## Discussion

After tooth extraction, the surrounding hard and soft tissues undergo physiological and structural changes that may jeopardize their integrity and volume, which is critical for any subsequent implant rehabilitation. The peri-implant mucosa needs to be supported by an adequate three-dimensional osseous volume to achieve functional and esthetic success [16].

According to Araujo et al. (2013), the extent of bone loss following tooth extraction depends on factors such as the bone wall thickness, position and angulation of the tooth, presence of surgical trauma, the flap elevation, a lack of functional stimulus and of the periodontal ligament as well. Regarding the bone thickness, animal and human studies have shown that the facial bone wall, especially in anterior maxilla sites, can be less than 1 mm in 90% of cases and less than 0.5 mm in almost 50% of cases. Thus, the reason for the significant loss after extraction observed in some cases may be because thin bone walls mainly consist of bundle bone, a lamellar bone structure with a thickness of 0.2–0.4 mm depending on the presence of the tooth and the blood supply from the periodontal ligament [17–19].

By contrast, thick facial bone wall and the palatal/lingual plates of teeth undergo minor changes. Although molar sites show larger ridge reductions, it is more critical to treat the anterior maxillary region because it has a larger impact on esthetics and the quality of the bone is lower [20, 21].

To counteract physiological bone loss, alveolar ridge preservation techniques that use autologous bone or biomaterial grafting and an additional absorbable membrane have been widely applied. According to recent clinical trials and systematic reviews, although socket grafting cannot prevent the resorption of facial and palatal bone wall, it helps to preserve the volume of the bone and leads to better clinical and histological outcomes [22–24].

A recent clinical study by Lee et al. (2021) tested the feasibility of implant placement in periodontally compromised patients treated either with ARP or with SH: the results showed that ARP can ease the implant placement and reduce the quantity of bone augmentation required [25].

For some important indications, alveolar ridge preservation procedures might be a valid alternative not only to spontaneous healing but also to other procedures such as the immediate implant placement or type 1 implants, according to the consensus statement by Tonetti et al. [11]: younger or systemically compromised patients, post-extractive sites in which it is not possible to achieve primary stability, and proximity to important anatomical structures (i.e., maxillary sinus, inferior alveolar nerve). Furthermore, ARP may be a predictable treatment choice for patients who are unable to undergo implant placement and desire to wait for longer periods than usual to preserve the architecture in an esthetic area and to avoid more invasive procedures in the subsequent phases, such as bone augmentation or sinus-lifting procedures [26].

The present study compared the dimensional changes at 6 months post-treatment between an extraction site treated with ARP and an alveolus treated with unassisted healing: the null hypothesis of no difference in the alveolar ridge and soft tissue changes between spontaneous healing and ARP was rejected and the results obtained using a bovine xenograft covered with an absorbable bovine pericardium membrane were comparable with those of Jung et al., who used xenografts [15]. In that study, the group treated with demineralized bovine bone mineral with either a soft tissue graft or a collagen matrix showed less vertical and horizontal change than the test group treated with beta-tricalcium phosphate and the control group who underwent spontaneous healing.

The ARP in our study was performed both flapless and with the elevation of a muco-periosteal flap, which was carried out only when required due to the complexity of the extraction. The influence of flap design on improving healing is much debated. According to more recent clinical studies, the surgical trauma, which includes raising a muco-periosteal flap with vertical incision and the detachment of periosteum, may lead to an extra amount of bone loss from the external part of the socket in addition to the bone resorption determined by the loss of bundle bone from the inside [27–30].

The systematic review by Vignoletti et al. [31] identified the wound closure technique as the most important factor influencing the outcome of ARP. In contrast, a more recent systematic review by Lee et al. [32] investigated the effects of different ARP procedures and found no significant differences in alveolar ridge height and width between the technique performed with the flap, the flapless technique with the application of a membrane, and the adjunctive use of a free gingival graft. Notably, compared with secondary closure, primary wound closure did not have a positive effect on preservation. It had a negative effect because it can result in greater shrinkage of the keratinized gingiva.

The optimal type of graft remains unclear. Bone grafting materials used for alveolar ridge preservation can be autogenous, allografts, xenografts, or alloplasts. Each biomaterial has specific features such as the resorption rate, which can be very slow for alloplasts and xenografts but faster for autogenous bone and allografts [33].

The use of a barrier membrane has also been investigated, and recent studies have found that the combination of a biomaterial and an absorbable membrane can succeed in decreasing both horizontal and vertical ridge shrinkage [34].

The review by Jung et al. (2018) examined ARP procedures for soft tissue and hard tissue preservation. For soft tissue preservation, the options available are autogenous connective tissue graft from tuberosity or palate or soft-tissue substitutes in order to reduce postoperative discomfort. For hard tissue preservation, the most documented procedure was the combination of a bone substitute material covered with an absorbable membrane. Although various materials have been used for ARP procedures, no one material has been found to be better than the others [35].

The systematic review by Avila et al. (2019) evaluated the effect of different ARP treatment modalities by analyzing clinical, radiographic, and patient-reported outcomes. The authors concluded that ARP procedures could prevent horizontal, vertical mid-buccal, and vertical mid-lingual bone resorption, but it was not possible to assess the superiority of one procedure over another [36].

A clinical trial by Papace et al. (2021) compared the use of autologous connective tissue graft with a collagen matrix for soft tissue management in ARP: they resulted to be comparable in terms of gingival thickness and peri-implant health [37].

The biomaterial used in our study was a bone substitute of bovine origin processed at low temperatures and able to create an environment favorable to osteoblast proliferation and bone regeneration. The main advantage of this biomaterial is its osteoinductive property due to the preservation of most of the bone cell matrix proteins. These proteins can induce specific bone markers involved in bone regeneration, such as osteopontin, osteocalcin, osteonectin, and type 1 collagen, as has been demonstrated in in vitro experiments. Moreover, its microporosity allows vessels and cells to colonize the graft so that its resorption time is reduced.

Regarding the width and height of the alveolar ridge, our findings are in line with those of previous studies that found that ARP techniques cannot fully preserve them. A certain amount of bone resorption should be expected when implementing ARP procedures, although there will be less resorption than if the alveolus was left to heal spontaneously [4].

The bone loss that occurred at the extraction sites in the control group was similar to that reported in the systematic review by Tan et al. [38]. That review stated that after tooth extraction, the percentage of vertical buccal bone resorption was 11–22% (0.8–1.5 mm, weighted average 1.24 mm at 6 months), and the percentage of horizontal buccal bone resorption was 29–63% (2.46–4.56 mm, weighted mean 3.79 mm at 6 months), with vertical resorption being less pronounced than horizontal resorption at 6 months.

The anterior superior esthetic zone is considered to be an area at high risk of alteration following tooth extraction and can experience marked changes in the surrounding soft tissue. Adequately supporting the soft tissue during ARP procedures is crucial for esthetics, and it may decrease the need for further soft tissue grafting in the future [39].

This study used digital 3D models of the dental arches to analyze the soft tissue independent of bone quantification.

The mean buccal soft tissue loss over a 6-month period after tooth extraction was 62.66 mm^3^ ± 17.50 mm^3^ for the ARP group and 106.41 ± 24 mm^3^ for the SH group, which was a significant difference (*P* = 0.004).

The results of the present study indicate that a reduction in the buccal soft tissue volume is expected following tooth extraction in which the alveolus is left to heal spontaneously. The interpretation of these data necessarily requires caution because the soft tissue volume loss was measured independently of the volume of the underlying bone resorption, and its clinical significance requires a different interpretation in relation to the linear measurements reported in millimeters.

Because of the high heterogeneity among studies in terms of morphology, biomaterials, surgical techniques, and healing periods, caution is needed when comparing our results with those of previous studies. Our study was a retrospective analysis of a small sample who were treated without a standardized protocol. Moreover, our study did not utilize randomization or histological analysis, which could have helped to evaluate the quality of the vital regenerated bone and its proportions. Nevertheless, the comparison of the ARP and SH groups offers an analysis of many factors that might positively influence ARP procedures, such as the preoperative evaluation of FSTT, analyzing the buccal bone thickness with CBCT, assessing the effect of a flapless approach when possible, and examining the combination of a xenograft plus an absorbable membrane. Moreover, the results were evaluated using linear measurements of CBCT and volumetric measurements of the changes in the region of interest to analyze the soft tissue profile. Finally, patient-reported outcomes and professional esthetic analysis with the Pink Esthetic Score were reported at 1 year post-treatment.

## Conclusion

In the present study, significant horizontal and vertical bone loss was observed after extraction in both groups. The group comparison revealed significantly larger vertical and horizontal bone reduction and larger volumetric shrinkage in the spontaneous healing group compared with the ARP group. Furthermore, ARP was associated with an increased PES and a reduction in the need for additional grafting procedures in subsequent treatment phases.

## Data Availability

The datasets used and/or analyzed during the current study are available from the corresponding author on reasonable request.

## References

[1] Gerritsen AE, Allen PF, Witter DJ, Bronkhorst EM, Creugers NH. Tooth loss and oral health-related quality of life: a systematic review and meta-analysis., Health Qual Life Outcomes, 8: 126 10.1186/1477-7525-8-126 PMC2992503 21050499

[2] Araujo M, Linder E, Wennström J, Lindhe J (2008). The influence of Bio-Oss collagen on healing of an extraction socket: an experimental study in the dog. Int J Periodont Restor Dent.

[3] Araujo MG, Lindhe J (2005). Dimensional ridge alterations following tooth extraction: an experimental study in the dog. J Clin Periodontol.

[4] Schropp L, Wenzel A, Kostopoulos L, Karring T (2003). Bone healing and soft tissue contour changes following single-tooth extraction: a clinical and radiographic 12-month prospective study. Int J Periodont Restor Dent.

[5] Seibert JS, Salama H (2000). Alveolar ridge preservation and reconstruction. Periodontol.

[6] Artzi Z, Nemcovsky CE (1998). The application of deproteinized bovine bone mineral for ridge preservation prior to implantation: clinical and histological observations in a case report. J Periodontol.

[7] Hammerle CH, Araujo MG, Simion ME (2012). Evidence-based knowledge on the biology and treatment of extraction sockets. Clin Oral Implants Res.

[8] Horvath A, Mardas N, Mezzomo LA, Needleman IG, Donos N (2013). Alveolar ridge preservation. A systematic review. Clin Oral Investig.

[9] Darby I, Chen ST, Buser D (2009). Ridge preservation techniques for implant therapy. Int J Oral Maxillofac Implants.

[10] De Risi V, Clementini M, Vittorini G, Mannocci A, De Sanctis M (2015). Alveolar ridge preservation techniques: a systematic review and meta-analysis of histological and histomorphometrical data. Clin Oral Implants Res.

[11] Tonetti MS, Jung RE, Avila-Ortiz G (2019). Management of the extraction socket and timing of implant placement: consensus report and clinical recommendations of group 3 of the XV European Workshop in Periodontology. J Clin Periodontol.

[12] Araujo MG, Lindhe J (2005). Dimensional ridge alterations following tooth extraction. An experimental study in the dog. J Clin Periodontol.

[13] Avila-Ortiz G, Chambrone L, Vignoletti F (2019). Effect of alveolar ridge preservation interventions following tooth extraction: a systematic review and meta-analysis. J Clin Periodontol.

[14] Avila-Ortiz G, Elangovan S, Kramer KWO, Blanchette D, Dawson DV (2014). Effect of alveolar ridge preservation after tooth extraction: a systematic review and meta-analysis. J Dent Res.

[15] Jung RE, Philipp A, Annen BM (2013). Radiographic evaluation of different techniques for ridge preservation after tooth extraction: a randomized controlled clinical trial. J Clin Periodontol.

[16] Cairo F, Nieri M, Cavalcanti R, Landi L, Rupe A, Sforza NM, Pace R, Barbato L (2020). Marginal soft tissue recession after lateral guided bone regeneration at implant site: a long-term study with at least 5 years of loading. Clin Oral Implants Res.

[17] Araújo MG, Silva CO, Misawa M, Sukekava F (2015). Alveolar socket healing: what can we learn?. Periodontol 2000.

[18] Januário AL, Duarte WR, Barriviera M, Mesti JC, Araújo MG, Lindhe J (2011). Dimension of the facial bone wall in the anterior maxilla: a cone-beam computed tomography study. Clin Oral Implants Res.

[19] Huynh-Ba G, Pjetursson BE, Sanz M, Cecchinato D, Ferrus J, Lindhe J, Lang NP (2010). Analysis of the socket bone wall dimensions in the upper maxilla in relation to immediate implant placement. Clin Oral Implants Res.

[20] Pietrokovski J, Massler M (1967). Alveolar ridge resorption following tooth extraction. J Prosthet Dent.

[21] Meijndert CM, Raghoebar GM, Vissink A, Meijer HJA (2021). Alveolar ridge preservation in defect sockets in the maxillary aesthetic zone followed by single-tooth bone level tapered implants with immediate provisionalization: a 1-year prospective case series. Int J Implant Dent.

[22] Araújo MG, da Silva JCC, de Mendonça AF, Lindhe J (2015). Ridge alterations following grafting of fresh extraction sockets in man. A randomized clinical trial. Clin Oral Implants Res.

[23] Chappuis V, Araújo MG, Buser D (2017). Clinical relevance of dimensional bone and soft tissue alterations post-extraction in esthetic sites. Periodontol 2000.

[24] Roberto C, Paolo T, Giovanni C, Ugo C, Bruno B, Giovanni-Battista MF (2021). Bone remodeling around implants placed after socket preservation: a 10-year retrospective radiological study. Int J Implant Dent.

[25] Lee J, Yun J, Kim JJ, Koo KT, Seol YJ, Lee YM (2021). Retrospective study of alveolar ridge preservation compared with no alveolar ridge preservation in periodontally compromised extraction sockets. Int J Implant Dent.

[26] Morton D, Chen ST, Martin WC, Levine RA, Buser D (2014). Consensus statements and recommended clinical procedures regarding optimizing esthetic outcomes in implant dentistry. Int J Oral Maxillofac Implants.

[27] Jonker BP, Gil A, Naenni N, Jung RE, Wolvius EB, Pijpe J (2021). Soft tissue contour and radiographic evaluation of ridge preservation in early implant placement: a randomized controlled clinical trial. Clin Oral Impl Res.

[28] Aimetti M, Manavella V, Corano L, Ercoli E, Bignardi C, Romano F (2018). Three-dimensional analysis of bone remodeling following ridge augmentation of compromised extraction sockets in periodontitis patients: a randomized controlled study. Clin Oral Impl Res.

[29] Wood DL, Hoag PM, Donnenfeld OW, Rosenfeld LD (1972). Alveolar crest reduction following full and partial thickness flaps. J Periodontol.

[30] Heitz-Mayfield LJA, Trombelli L, Heitz F, Needleman I, Moles D (2002). A systematic review of the effect of surgical debridement vs non-surgical debridement for the treatment of chronic periodontitis. J Clin Periodontol.

[31] Vignoletti F, Matesanz P, Rodrigo D, Figuero E, Martin C, Sanz M (2012). Surgical protocols for ridge preservation after tooth extraction. A systematic review. Clin Oral Impl Res.

[32] Lee J, Lee J-B, Koo K-T, Seol Y-J, Lee Y-M (2018). Flap management in alveolar ridge preservation: a systematic review and meta-analysis. Int J Oral Maxillofac Implants.

[33] Al Yafi F, Alchawaf B, Nelson K (2019). What is the Optimum for Alveolar Ridge Preservation?. Dent Clin North Am.

[34] Troiano G, Zhurakivska K, Lo Muzio L, Laino L, Cicciù M, Lo RL (2018). Combination of bone graft and resorbable membrane for alveolar ridge preservation: a systematic review, meta-analysis, and trial sequential analysis. J Periodontol.

[35] Jung RE, Ioannidis A, Hämmerle CHF, Thoma DS (2018). Alveolar ridge preservation in the esthetic zone. Periodontol 2000.

[36] Avila-Ortiz G, Chambrone L, Vignoletti F (2019). Effect of alveolar ridge preservation interventions following tooth extraction: a systematic review and meta-analysis. J Clin Periodontol.

[37] Papace C, Büsch C, Ristow O, Keweloh M, Hoffmann J, Mertens C (2021). The effect of different soft-tissue management techniques for alveolar ridge preservation: a randomized controlled clinical trial. Int J Implant Dent.

[38] Tan WL, Wong TLT, Wong MCM, Lang NP (2012). Systematic review of post extractional alveolar hard and soft tissue dimensional changes in humans. Clin Oral Impl Res.

[39] Chappuis V, Engel O, Shahim K, Reyes M, Katsaros C, Buser D. Soft tissue alterations in esthetic postextraction sites: a 3-dimensional analysis. J Dent Res. 2015; 94 (9 Supp). 10.1177/0022034515592869 26130259

